# Sarcopenic Obesity Predicts Early Attrition in Treatment-Seeking Patients with Obesity: A Longitudinal Pilot Study

**DOI:** 10.3390/jcdd7010005

**Published:** 2020-01-27

**Authors:** Dima Kreidieh, Leila Itani, Hana Tannir, Dana El Masri, Marwan El Ghoch

**Affiliations:** Department of Nutrition and Dietetics, Faculty of Health Sciences, Beirut Arab University, P.O. Box 11-5020 Riad El Solh, Beirut 11072809, Lebanon; d.kraydeyeh@bau.edu.lb (D.K.); l.itani@bau.edu.lb (L.I.); hana.tannir@bau.edu.lb (H.T.); dana.masri@bau.edu.lb (D.E.M.)

**Keywords:** BMI, obesity, sarcopenic obesity, dropout, attrition, outcome, body composition

## Abstract

Attrition is a major cause of failure in obesity treatment, which is still not fully understood. The identification of factors related to this outcome is of clinical relevance. We aimed to assess the relationship between sarcopenic obesity (SO) and early attrition. Early attrition was assessed at six months, and two groups of patients were selected from a large cohort of participants with overweight or obesity enrolled at the Outpatient Clinic of the Department of Nutrition and Dietetics at Beirut Arab University (Lebanon). Body composition was measured using a bioimpedance analyser (Tanita BC-418) and participants at baseline were categorized as having or not having SO. The “dropout group” included 72 participants (cases) compared to 31 participants (controls) in the “completer group”, with the former displaying a higher prevalence of SO than the latter (51.0% vs. 25.8%; p = 0.016). In the same direction, Poisson regression analysis showed that SO increased the relative risk of dropout by nearly 150% (RR = 1.45; 95% CI = 1.10–1.89; p = 0.007) after adjustment for age, gender, body mass index (BMI), age at first dieting, sedentary habits and weight-loss expectation. In conclusion, in a “real-world” outpatient clinical setting, the presence of SO at baseline increases the risk of dropout at six months. New directions of future research should be focused on identifying new strategies to reduce the attrition rate in this population.

## 1. Introduction

In 2018, the European Society for Clinical Nutrition and Metabolism (ESPEN) and the European Association for the Study of Obesity (EASO) declared that sarcopenic obesity (SO) [1,2,3,4,5,6]—which is represented by the coexistence of obesity (i.e., increase in body fat mass deposition) [7,8] and sarcopenia (i.e., decrease in muscle mass and strength) [9]—should be considered a priority by both researchers and clinicians [10]. The reason behind this recommendation stems from the fact that patients with SO appear to have a higher risk of cardiometabolic diseases as well as psychosocial comorbidities when compared to their counterparts without SO [1,2,3,11,12].

In the same direction, preliminary findings recently evidenced that SO is associated with a reduction in energy expenditure (e.g., resting metabolic rate) [13]. This opened up new directions in research which aims to determine whether this disadvantaged metabolic phenomenon (e.g., resting metabolic rate) in individuals with SO may have, in some way, a negative impact on clinical outcomes (e.g., attrition, weight loss or maintenance) during weight management programmes [4]. 

This being said, attrition is one of the major causes of failure on weight management programmes for obesity, with rates that range between 10% and 80% according to the type (e.g., lifestyle modification programmes, medication, bariatric surgery) and the design of the treatment (e.g., randomized trails, observational studies) [14,15,16,17]. Several anthropometric, sociodemographic and psychosocial factors have been identified as being related/associated with higher rates of dropout [14,15,16,17,18,19]. However, attrition during obesity treatment is complex and has not been fully understood. Therefore, the identification of new factors that lead to premature programme termination and the implementation of effective strategies to prevent the latter are needed to reduce attrition rates, considered vital in ensuring long-term success in weight management program.

In light of these considerations, the current study aimed to investigate the relationship between SO and early attrition rates in a “real-world” clinical setting of treatment-seeking patients with overweight or obesity, using a definition of SO that, in addition to appendicular skeletal muscle mass (ASM), also includes body weight, namely the definition proposed by Oh and colleagues [20], which in previous studies has been demonstrated to be of higher clinical value in our population than other definitions [1,13].

## 2. Materials and Methods

Participants were selected from a cohort of 184 participants consecutively admitted to the Outpatient Clinic of the Department of Nutrition and Dietetics at Beirut Arab University (Lebanon) for a weight management programme for the treatment of obesity from May 2017 to May 2019. Patients were considered eligible if they were aged ≥ 18 years, with a BMI ≥ 25.0 kg/m^2^ and at least one of a number of weight-related comorbidities (e.g., type 2 diabetes, cardiovascular disease, sleep apnoea, severe joint disease, etc.), as well as if they were identified as suitable for weight-loss treatment and effectively started the treatment. A total of 72 of the 145 patients assessed for eligibility was included because they met the following conditions: they (i) were effectively enrolled on the programme and (ii) interrupted treatment during the weight-loss phase (before six months). Based on these cases (N = 72), 31 controls were selected from the same cohort, all of which had a similar BMI and the same gender to form a comparison group with a 2:1 ratio, as well as having successfully completed the weight-loss phase (six months). The programme featured a low-calorie diet and the protocol for the treatment essentially involved a personalized cognitive behavioural treatment (CBT-OB) programme designed for patients with obesity, as described elsewhere [21,22]. The study was approved by the Institutional Review Board of Beirut Arab University (No. 2017H-0034-HS-R-0241), and all participants provided informed written consent.

### 2.1. Demographics and Clinical Status

A questionnaire was administered to participants in order to retrieve information regarding their medical history, sociodemographic and clinical status (age, marital status, employment, level of education, age at first dieting, dietary and lifestyle habits, weight-loss expectation).

### 2.2. Baseline Measures

A questionnaire was administered that retrieved information about the medical history, demographic, social, information (e.g., age, gender, marital status, employment and education) and other factors known to be associated with higher attrition rates, identified from the available literature (i.e., body mass index (BMI)), age at first dieting, sedentary habits and weight-loss expectation.

Body weight and height were measured using an electronic weighing scale (SECA 2730-ASTRA, Germany) and a stadiometer. The BMI was then calculated according to the standard formula of body weight measured in kilogrammes, divided by the square of the height in metres. 

Body composition was measured using a segmental body composition analyser (BC-418, Tanita Corp., Tokyo, Japan) [23]. After the gender, age and height information had been entered into the device, participants were asked to stand bare feet in a stable position. The device provided separate body mass readings for different segments of the body, using an algorithm incorporating impedance, age and height, to estimate the total and regional fat mass (FM) and fat-free mass (FFM) [23,24,25]. SO was defined based on the definition of Oh and colleagues; that is, a score of less than 23.4 in females and 29.6 in males using the formula ASM/weight × 100% [20].

### 2.3. Six-Month Measures

Early attrition was assessed at six months by analysing the medical records, where the date of the last visit when patients were last seen were registered.

### 2.4. Statistical Analysis

The normality of the data was checked using Shapiro–Wilk or Kolmogorov–Smirnov tests, as well as quintile plots. The normality checks revealed unacceptable normality; hence, non-parametric tests were used for comparison. Frequencies, medians and interquartile ranges were used to describe the anthropometric characteristics of the study population. Medians and frequencies were compared using the Mann–Whitney U test and the chi-squared test, respectively. Poisson regression was used to assess the association between dropout events and SO as an independent variable, while also adjusting for other covariates, including age, gender baseline BMI (kg/m^2^), sedentary habits and weight-loss in kg expectation over 12 months. All analyses were performed using SPSS version 26.0 (IBM Corp.; IBM, Armonk, NY, USA). Tests were considered statistically significant at *p* < 0.05.

## 3. Results

Table 1 presents the sociodemographic and clinical characteristics of the patients included in the study. The median age of the total study sample was 35 (IQR = 26.44) years, with patients in the dropout group being younger (31.15, IQR = 25.16) than those in the completers group (47.45, IQR = 17.11). Almost two thirds of the sample comprised females (69.9%) with a similar proportion among dropouts (65.3%) and completers (80.6%) (*p* = 0.119). Both groups had a similar median baseline BMI (35.06, IQR = 7.10 vs. 34.19, IQR = 6.20) and weight-loss expectations in kg over 12 months (20.00, IQR = 13.00 vs. 15.00, IQR = 10.00). Dropout patients apparently started dieting at a younger age (19.00, IQR = 9.00) compared to completers (25.00, IQR = 20.50). Both the dropout patients and the completers did not differ in terms of marital status, education, employment and sedentary habits.

The overall prevalence of SO in the study sample was 43.7% with a higher prevalence among dropouts (51.4%) compared to completers (25.8%). Similarly, a higher dropout rate (82.0% vs. 60.0%) was observed among those patients with SO. Furthermore, Poisson regression analysis showed that SO increased the relative risk of dropout by nearly 150% (RR = 1.45; 95% CI = 1.10–1.89; *p* = 0.007) while controlling for age, gender, baseline BMI, age at first dieting, sedentary habits and weight-loss expectation (Table 2).

## 4. Discussion

Our study aimed to provide preliminary data on the relationship between SO and treatment outcomes, namely, the attrition rate in adults with overweight or obesity. In turn, one major finding was revealed.

The group of participants who dropped out had a higher proportion of SO compared to their counterparts who completed the six-month weight-loss phase (51.0% vs. 25.8%; *p* = 0.016). In fact, the presence of SO increased the risk of dropout by nearly 150% compared with those without SO, while controlling for age, gender, baseline BMI, age at first dieting, sedentary habits and weight-loss expectation. To date, our study is the first to report such a finding in the literature; hence, it is difficult to compare it with previous studies conducted among this population. Moreover, the underlying mechanism behind this relationship is still unclear. However, we speculate that one of the reasons behind our finding could stem from the limited weight-loss rate in patients with SO due to the reduced energy expenditure when compared to those without SO, especially if a higher weight-loss expectation is an independent dropout predictor, as was reported in recent studies [18,26,27]. Therefore, future more studies focusing on treatment outcomes (i.e., weight-loss rates, weight maintenance) in the case of SO are needed if firm conclusions are to be drawn.

Our study has certain strengths. Principally, to the best of our knowledge, it is the first one to assess the relationship between attrition rates and SO in treatment-seeking patients with overweight or obesity. Furthermore, the longitudinal design and the “real-world” clinical setting of the study should be considered as strengths. However, our study did have some limitations. First, our sample included only patients seeking an outpatient weight management treatment program; hence, our findings are not extendable to patients with obesity who seek other treatment modalities (e.g., bariatric surgery, pharmacological interventions, etc.). Secondly, we assessed body composition using an impedance analyser, which, despite being validated, has still not been accepted as the gold-standard technique for patients with overweight and obesity [24]. Thirdly, the use of a definition for SO that was initially established in an Asian population [20], which was based only on a reduction in lean body mass (LBM) and thus not taking into account low muscle strength or low physical function, should be considered a further limitation. Fourthly, we did not take into consideration other factors that may influence treatment attrition, such as psychometric assessments to detect the presence/absence of depression that can be a confounder and thus introduce bias during data interpretation. Finally, due to the relatively small sample size, these results are preliminary and need further replication. If confirmed, our finding may have relevant clinical implications for targeting patients with SO where there is a higher risk of dropout, as implementing additional strategies for this subgroup of patients may be useful in reducing treatment attrition.

## 5. Conclusions

In our study, we provide evidence that SO leads to a higher risk of dropout. Undoubtedly, this finding needs to be replicated using larger samples and, if confirmed, provides a new direction for future studies seeking to determine the impact of this phenotype (i.e., SO) on clinical outcomes (i.e., having difficulties losing or maintaining weight), as well as emphasizing the importance of developing further strategies for these patients regarding weight management programs.

## Figures and Tables

**Table 1:** Socio demographic and anthropometric characteristics of the study population (N = 103) *.

	Total (N = 103)	Dropouts (N = 72)	Completers (N = 31)	Significance
Age (Years)	35.07(26.44)	31.15(25.16)	47.45(17.11)	*p* = 0.002
Gender				X^2^ = 2.432; *p* = 0.119
Male	31(30.1)	25(34.7)	6(19.4)	
Female	72(69.9)	47(65.3)	25(80.6)	
Marital status				X^2^ = 2.203; *p* = 0.138
Not married	48(46.6)	37(51.4)	11(35.5)	
Married	55(53.4)	35(48.6)	20(64.5)	
Employment				X^2^ = 0.713; *p* = 0.398
Not employed	60(58.3)	40(55.6)	20(64.5)	
Employed	43(41.7)	32(44.4)	11(35.5)	
Education				X^2^ = 0.713; *p* = 0.398
Lower education	60(58.3)	40(55.6)	20(64.5)	
Higher education	43(41.7)	32(44.4)	11(35.5)	
Baseline BMI (kg/m^2^)	34.91(6.81)	35.06(7.10)	34.19(6.20)	*p* = 0.392
Age at first dieting	20.50(12.00)	19.00(9.00)	25.00(20.50)	*p* = 0.033
Sedentary habits				X^2^ = 1.012; *p* = 0.798
Very sedentary	22(21.4)	17(23.6)	5(16.1)	
Sedentary	25(24.3)	16(22.2)	9(29.0)	
Active	42(40.8)	29(40.3)	13(41.9)	
Very active	14(13.6)	10(13.9)	4(12.9)	
Weight-loss expectation in 12 months (kg)	17.00(14.00)	20.00(13.00)	15.00(10.00)	*p* = 0.113
Presence of SO				X^2^ = 5.765; *p* = 0.016
No	58(56.3)	35(48.6)	23(74.2)	
Yes	45(43.7)	37(51.4)	8(25.8)	

* The Values are medians (IQR = interquartile range) for continuous variables and (n%) for categorical variables; BMI = body mass index; SO = sarcopenic obesity.

**Table 2:** Relative risk of dropout among patients with SO (N = 103).

	RR	95%CI
Age (years)	0.99	0.97–1.00
Gender		
Males	1	
Females	0.87	0.68–1.12
Baseline BMI (kg/m^2^)	1.01	0.99–1.04
Age at first dieting	0.99	0.97–1.02
Sedentary habits		
Very sedentary	1	
Sedentary	0.83	0.55–1.24
Active	1.11	0.76–1.62
Very active	1.01	0.59–1.73
Weight-loss expectation in 12 months	1	0.98–1.01
SO		
No	1	
Yes	1.45	1.10–1.89

* BMI = body mass index; SO = sarcopenic obesity.
